# Photodynamic Therapy of Presumed Choroidal Metastasis Secondary to Colorectal Carcinoma: Literature Review

**DOI:** 10.1155/2020/6490535

**Published:** 2020-01-30

**Authors:** Laurentino Biccas Neto, José Z. Pulido, Gustavo B. Melo, Luiz H. Lima, Eduardo B. Rodrigues

**Affiliations:** ^1^Ocular Oftalmologia, Vitória, Brazil; ^2^Department of Oncology, Hospital Evangélico, Cachoeiro do Itapemirim, Brazil; ^3^Hospital de Olhos de Sergipe, Aracaju, Brazil; ^4^Department of Ophthalmology, Universidade Federal de São Paulo, São Paulo, Brazil; ^5^Department of Ophthalmology, Saint Louis University School of Medicine, Saint Louis, MO, USA

## Abstract

Colorectal cancer may yield metastasis to the choroid. Its management may be challenging, since there is no consensus about treatment. We describe a case of a 70-year-old male with colon cancer who complained of worsening visual acuity of his better-seeing eye to 20/40 secondary to a nonpigmented choroidal mass of medium reflectivity under the inferior temporal arcade and neurosensory foveal detachment. Besides systemic chemotherapy, local treatment with verteporfin photodynamic therapy (vPDT) was performed. After one month, visual acuity improved to 20/25 and subretinal fluid faded. In conclusion, vPDT may be a useful adjuvant treatment modality for choroidal metastasis secondary to colorectal cancer.

## 1. Introduction

Colorectal cancer (CRC) consists of the development of cancer from the colon or rectum. Various histology types of CRC have been described, whereas adenocarcinoma comprises over 95% of the cases. About 20% of the patients with CRC present with metastases at diagnosis, and the most common sites include the liver, lung, peritoneum, and bones [1–3].

Ocular and periocular metastases in patients with CRC have been described in the medical literature, with the choroid being the main site [4–8]. Choroidal metastasis may be uni- or bilateral and may comprise around 4% of CRC sites of metastasis [8, 9]. Management of such choroidal lesions may be challenging, since there is no consensus about treatment. It might include observation, radiotherapy, or other modalities of local therapy, like vascular endothelial growth factor inhibitors.

The primary purpose of this paper was to report on a case of presumed choroidal metastasis in a patient with colon carcinoma treated by photodynamic therapy with verteporfin (vPDT). The secondary goal of the paper was to present a review of medical literature about epidemiology, clinical features, and multimodal imaging findings, with special emphasis on the management options of choroidal and retinal metastasis secondary to CRC.

## 2. Case Report

**Figure 1:**
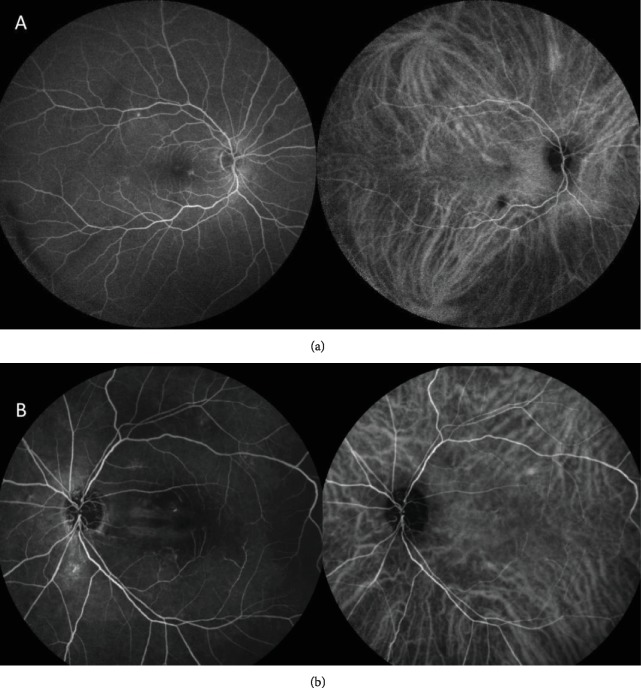
Images 5 months before the diagnosis of the metastatic lesion in the right eye (RE). (a) Midphase wide field FA and ICG of the RE showing discrete hyperfluorescent spots (transmission defects) in the arcades. Hypofluorescent plaque on ICG in the inferior temporal vascular arcade, with no relevant findings on multimodal OCT. (b) Late arterial phase FA and ICG of the left eye showing narrow arterial vessels and horizontal hyperfluorescent tracks due to RPE atrophy in the central macula.

A 70-year-old male was referred with a complaint of complete and sudden visual loss in the left eye (LE) immediately following a cardiac surgery (mitral valve exchange). The patient informed previous diagnosis of a spinal lesion following a spinal cord abscess on T9 which led to paraplegy. On ophthalmic examination, best-corrected visual acuity (BCVA) was 20/20 in the right eye (RE) and no light perception in the LE, which showed a relative afferent pupillary defect (RAPD). Intraocular pressure (IOP) was 15 mmHg in both eyes (BE). Multimodal imaging demonstrated acute central retinal artery occlusion in the LE; in the RE, there were discrete soft drusen throughout the macula. A small area of hypofluorescence was seen on ICG angiography near the inferior temporal vascular arcade in the RE but was deemed to be irrelevant (Figure 1). No intraocular masses were seen in the macular area on Spectral Domain OCT (SDOCT). Blue autofluorescence imaging was unremarkable in the RE.

**Figure 2:**
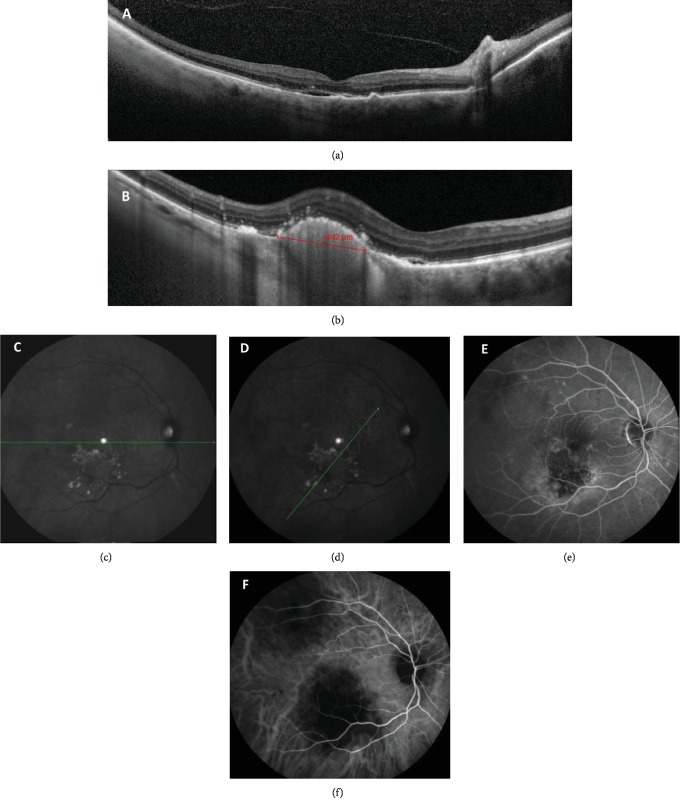
A metastatic lesion appears in the right eye five months later. (a) Horizontal OCT showing a small neurosensory macular detachment, with discrete thickening of the RPE and of the outer foveal lines over the detachment. Scattered hyperreflective foci within the retina. Note the loss of typical choroidal texture under the serous detachment. (b) OCT showing a choroidal mass with moderate internal reflectivity on OCT superior to the inferior temporal arcade, measuring 1,912 *μ*m in diameter, with no overlying choriocapillaris. Overlying hyperreflective foci and contiguous neurosensory foveal detachment are seen. (c, d) IR image depicts numerous whitish subretinal clumps suggesting fibrin along both vascular arcades. The green lines represent the axial orientation of the B-scans of images (a) and (b), respectively. (e) FA shows a hypofluorescent plaque surrounded by mottled hyperfluorescence in the inferior temporal arcade. (f) Hypofluorescent plaques on ICG along the superior and inferior vascular arcades.

Five months later, the patient returned to the ophthalmology office complaining of worsening of vision in the RE (better-seeing eye). In the meantime, he was diagnosed with colon adenocarcinoma at a metastatic stage (multiple lesions in the liver and lungs recently discovered) and treated with endovenous chemotherapy. The assistant oncologist informed that the chemotherapeutic regimen (bevacizumab+FOLFIRINOX) had led to a partial reduction of the systemic metastasis (estimated around 30%). FOLFIRINOX is a chemotherapy regimen for the treatment of advanced pancreatic cancer made up of the following four drugs: folinic acid (leucovorin), fluorouracil (5-FU), irinotecan, and oxaliplatin. At the second ophthalmic examination, BCVA was 20/40 in the RE and no light perception in the LE; anterior segment was normal in BE; there was a shallow neurosensory foveal detachment seen on SDOCT, along with a choroidal mass of medium reflectivity superior to the inferior temporal arcade in the RE (Figure 2). The choroidal lesion was nonpigmented, measuring 6 × 7 × 1.5 mm by ultrasound, without hemorrhages, hard exudates, or calcification. There was moderate pigment clumping and atrophy overlying the lesion, showing mottled areas of hypo- and hyperautofluorescence in the blue spectrum. Fundus examination of the LE revealed diffuse vascular attenuation and two parallel hyperfluorescent horizontal streaks in the macula as a result of the central arterial occlusion (unchanged from the previous examination 5 months before). On ICG, there were two new areas (compared to the previous exam) of hypofluorescence in the RE, one corresponding to the choroidal mass and a second one under the superior temporal arcade (Figure 2). SDOCT, matched to the superior hypofluorescent area, showed no evident tumor, but there was loss of the choroidal texture, as if no vessels could be seen in this layer.

Potential risks and benefits of verteporfin photodynamic therapy (vPDT) were discussed with the patient, and a signed informed consent was obtained. As the RE was the only seeing eye of the patient, it was decided that only the inferior lesion should be treated, fearing collateral damage by extensive vPDT in a single session. Laser settings and treatment guidelines of vPDT were performed according to the guidelines of the Treatment of Age-related Macular Degeneration with Photodynamic Therapy study protocol: standard full-fluence PDT was performed using a 6 mg/m^2^ dose of verteporfin and a 689 nm laser (Visulas 690S; Carl Zeiss Meditec, Inc.) with 50 J/cm^2^ of laser light delivered over a duration of 83 seconds on the area of the tumor. The spot size was adjusted to as wide as possible, in order to cover the largest diameter of the tumor with a 0.5 mm free margin. Three overlapping spots were necessary to cover the entire lesion.

**Figure 3:**
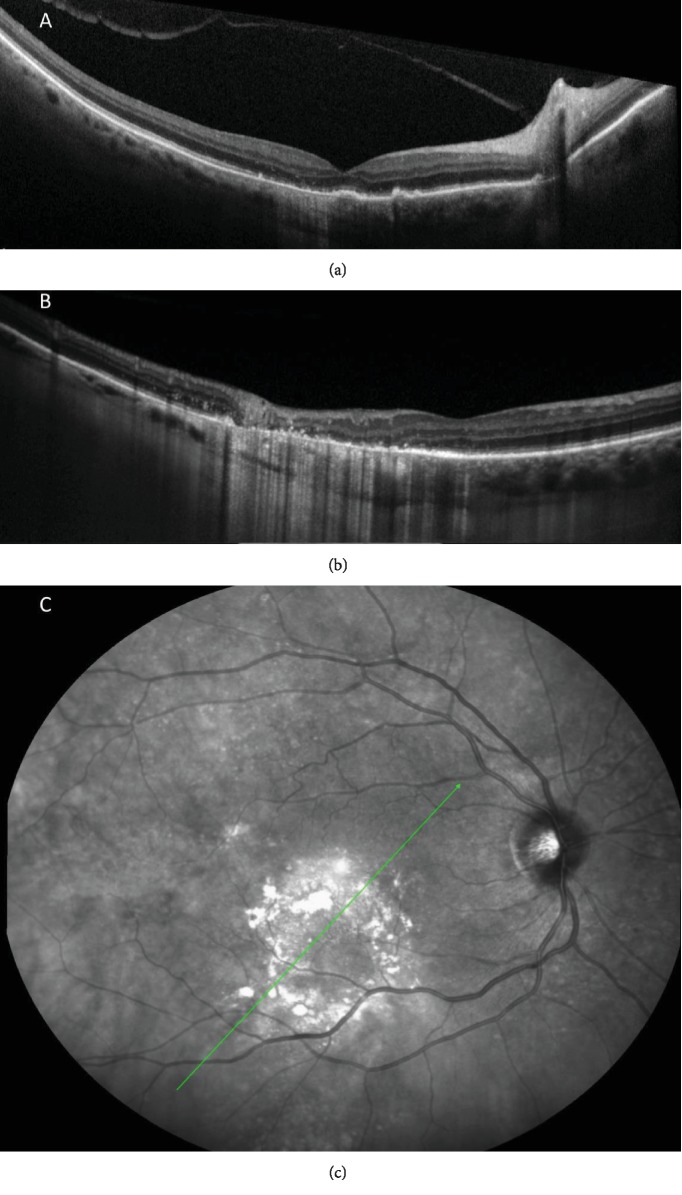
One month after photodynamic therapy. (a) Repeated OCT over the central macula (as seen in Figure 2(a)) showing complete resolution of the serous macular detachment. Some incomplete RPE and outer retinal atrophy (iRORA) are seen temporally along with irregularity and thickening of the RPE in the foveal region. (b, c) Coupled IR and OCT showing complete resolution of the choroidal mass under the inferior vascular arcade, with a broad complete RPE and retinal atrophy (cRORA) and subsidence of the inner retinal layers. No choroidal layer could be seen on this site. Green line (c) represents the axial orientation of the OCT scan in (b).

One month after vPDT, we observed a significant reduction in choroidal tumor thickness and in subretinal fluid. BCVA improved to 20/25 in the RE after successful vPDT. Fluorescein angiography disclosed a hypofluorescent plaque under the tumor region with mottled hyperfluorescent spots (Figure 3). ICG angiography revealed reduction in hyperfluorescent spots and enhancement in the hypofluorescent area. OCT over the tumor area showed an intraretinal dense hyperreflective band with underlying RPE irregularities and choroidal hyperreflectivity.

## 3. Main Text

Adenocarcinomas make up about 95% of CRC. Other less common tumors are lymphomas and sarcomas. The 5-year survival rate for patients with stage I colon cancer is about 90%, while those with a more advanced disease, such as in stage IV CRC, have an estimated survival rate of 10%. CRC may lead to a variety of distance metastases including the lung, liver, peritoneum, brain, and bones [1, 3, 10, 11]. Less common sites of metastasis secondary to CRC include the bladder, brain, adrenals, spleen, and the eye and its adnexa, whereas some isolated reports found metastatic disease in the orbit, periocular skin, retina, and choroid [4–7, 12–28]. Carcinoma can metastasize to the uvea from any portion of the colon, although the sigmoid colon and rectum appear to be most highly represented [19, 29].

Gastrointestinal metastases are relatively uncommon in the uvea [9, 29, 30]. This implies either a molecular property that makes the uvea particularly attractive to breast and lung cancers or a molecular difference between lung, breast, and gastrointestinal primary tumors that allows breast and lung cancers to metastasize more readily to the uvea.

The incidence of metastasis of CRC to the choroid and retina is not yet clear, even though a survey by Shields determined that 4% of all uveal metastases (43 eyes of 40 patients) are caused by tumors of the gastrointestinal tract in a survey of 2214 tumors in 1111 patients [8]. In 2014, Tei et al. [31] published one case of choroidal metastasis secondary to rectum carcinoma plus a review of thirteen case reports of the literature with a discussion about ophthalmic and systemic features. However, a deeper analysis on the therapeutic approaches for choroidal metastasis was not provided [31].

Details of the clinical features such as laterality and types of masses of choroidal metastasis can be obtained from case reports. Papers such as those published by Ma et al., Ha et al., Khawaja et al., and Lin et al. showed unilateral single choroidal mass in reports of colorectal adenocarcinoma [5, 13, 19, 32]. Moreover, Maudgil et al. described two eyes with a single choroidal mass in patients with diagnosis of adenocarcinoma of the cecum, while Ward et al. reported single bilateral choroidal masses in a patient with presumed colon cancer [33, 34]. Similar to most previous publications, our patient presented with a unilateral hyperreflective and hyperechogenic choroidal mass in the inferior temporal arcade. Shields et al. demonstrated that 93% of patients with choroidal metastasis due to tumors of the gastrointestinal tract have unilateral masses [8]. Based on this evidence, one may assume that a choroidal metastasis due to CRC will most likely induce a unilateral focal lesion compared to other types of tumors like breast carcinoma that present an average of 1.9 tumors per eye.

Only four cases of retinal metastases occurring in patients with CRC have been published in the medical literature so far to our knowledge [12, 35–37]. The lesions also were unilateral and solitary. Kennedy et al. described one case in 1956 in a male patient who showed a small-sized well-demarcated and grayish-white lesion in the macula extending into the vitreous. It was associated with annular carcinoma of the rectosigmoid junction. A second case was reported in a woman with Muir-Torre syndrome that had multiple tumors including sebaceous adenomas of the face and neck and adenocarcinoma of the colon and retinal tumor. She underwent enucleation of the eye for a retinal tumor and histopathology revealed adenocarcinoma. The case by Spraul et al. was of an adenocarcinoma of the cecum which led to an exudative retinal detachment. The retinal mass was resected by pars plana vitrectomy [36]. Nookala et al. proposed that when a patient experiences metastasis to the retina, there is a high chance of cancer spreading to the central nervous system, which may mean less favorable prognosis [12].

Treatment of symptomatic choroidal metastasis depends on the systemic status and staging of the metastatic disease. Options include observation (in preterminal patients or when metastasis regresses with chemotherapy), chemotherapy, immunotherapy, hormone therapy, radiotherapy, and even enucleation for blind and painful eyes harboring metastatic tumors [38]. Local palliative treatment of choroidal metastasis may be efficient and yield good control of visual symptoms in patients with a metastatic neoplasm with little collateral damage, as with vPDT.

Photodynamic therapy is a nonthermal laser modality primarily employed in the treatment of vascular diseases, through light activation of the photosensitizing agent verteporfin. This therapy may also be employed in the treatment of ocular tumors through induction of necrosis and apoptosis, damage to the intratumoral vasculature, and local inflammatory reaction [38]. vPDT seems to be more effective in treating retroequatorial tumors with low to moderate exudative symptoms and with a thickness less than 3 mm and diameter less than 10 mm. Lesions that are too thick might not be eligible for vPDT, since the wavelength (689 nm) of the laser used will not penetrate the entire tumor. Moreover, visual acuity seems to improve after vPDT probably due to the decrease of subretinal fluid [39].

There have been sporadic reports of vPDT for choroidal metastasis secondary to other sites, such as the breast, with good results [40–43]. In this study, we reported on a patient with presumed choroidal metastasis secondary to colorectal cancer who showed an excellent response after one session of vPDT. Due to the severity of the primary metastatic disease and the lack of effective therapies to eliminate the presumed metastatic lesions in the macula without considerable collateral damage, after a comprehensive discussion with the patient, we decided to employ vPDT as a palliative measure to assure useful vision to this patient's only seeing eye.

In addition to vPDT, other reports discussed intravitreal bevacizumab for local therapy of a choroidal mass. Intravitreal treatment might be a good therapeutic option for patients with a metastatic disease as it preserves the quality of life without reducing clinical effectiveness. Mathis et al. suggested beginning with a single dose followed by a monthly as-needed regimen [39]. It could be proposed for multimetastatic patients under systemic chemotherapy with short life expectancy, as it improves or preserves the vision in most cases without time-consuming treatment. Furthermore, unlike vPDT, this treatment can be given in cases of exudative or large tumors. In summary, local therapy of choroidal metastasis due to CRC may provide ancillary support and a vision-saving treatment option.

Prognosis of patients with choroidal metastasis is usually not favorable. In many patients, metastatic disease may be found elsewhere, associated with a low survival rate. A high index of suspicion must be considered in choroidal masses of unknown systemic malignancies. Systemic control of colon carcinoma may aid in the ocular tumor regression.

In summary, we presented a case of a patient with presumed choroidal metastasis secondary to colon adenocarcinoma. Local vPDT therapy was performed with marked reduction of the subretinal mass and vision improvement. Choroidal metastasis secondary to CRC may be usually unilateral, with a single mass, and associated with advanced systemic spread of the disease.
